# 1387. Impact of Cytomegalovirus Prophylaxis on Healthcare Resource Use and Costs among Kidney Transplant Recipients: A United States Renal Data System-Medicare Linked Database Study

**DOI:** 10.1093/ofid/ofab466.1579

**Published:** 2021-12-04

**Authors:** Amit D Raval, Michael Ganz, Priya Saravanan, Yuexin Tang, Carlos Santos

**Affiliations:** 1 Merck and Co., Inc., Rahway, New Jersey; 2 Evidera, Inc., Waltham, Massachusetts; 3 Merck and Co., Inc, North wales, Pennsylvania; 4 Rush University Medical Center, Chicago, Illinois

## Abstract

**Background:**

Cytomegalovirus (CMV) management requires a balance between reducing the risk of CMV infection and avoiding anti-viral toxicities. Limited information is available on the impact of CMV prophylaxis on the healthcare resource use (HCRU) and costs among adult kidney transplant recipients (KTRs) in the United States. Therefore, we examined HCRU and cost associated with CMV prophylaxis stratified by the CMV risk categories among KTRs at 1-year post-KT.

**Methods:**

We identified a cohort of 22,918 adults first-time KTRs during 2011–2017 using the US Renal Data System registry-linked Medicare data. Additional inclusion criteria were to have continuous coverage in Medicare Part A & B for ≥ 6-month pre- and ≥ 12-month post KT and Medicare Part D for ≥12-month post-KT. CMV prophylaxis was confirmed as ≥ 1 prescription fill for valacyclovir/(val)ganciclovir prophylaxis doses within 28 days post-KT.

**Results:**

CMV prophylaxis was utilized in 86%, 82%, and 32% of high, intermediate, and low-risk KTRs with an average cost of prophylaxis per KTRs of &16,241, &9481, and &8,648, respectively. In no prophylaxis groups, valganciclovir was utilized in 52%, 34%, and 36% of KTRs (as either pre-emptive or deferred therapy) with an average cost of &6,719, &2,722, and &431 among high, intermediate, and low-risk KTRs, respectively. Among high-risk KTRs, CMV prophylaxis group had a significantly higher prescription drug cost (&26,060 vs. &13,433) but a lower average direct healthcare medical cost (&84,914 vs. &101,268), mainly due to lower all-cause hospitalization cost (&56,758 vs. &69,852) (**Table 1**). CMV prophylaxis group had lower rates of all-cause rehospitalization, and CMV-and opportunistic infection (OIs)-related hospitalization compared to no prophylaxis (**Table 2**). In high-risk KTRs, nearly 32% had myelosuppressive events-related hospitalization, and 15% filled granulocyte colony-stimulating factors with an average cost of &4,695 per treated KTR.

**Conclusion:**

CMV prophylaxis had a higher cost of medications but had a lower medical cost with including all-cause and CMV-related hospitalizations. Myelosuppressive events were frequent and resource-intensive especially in high and intermediate-risk KTRs.

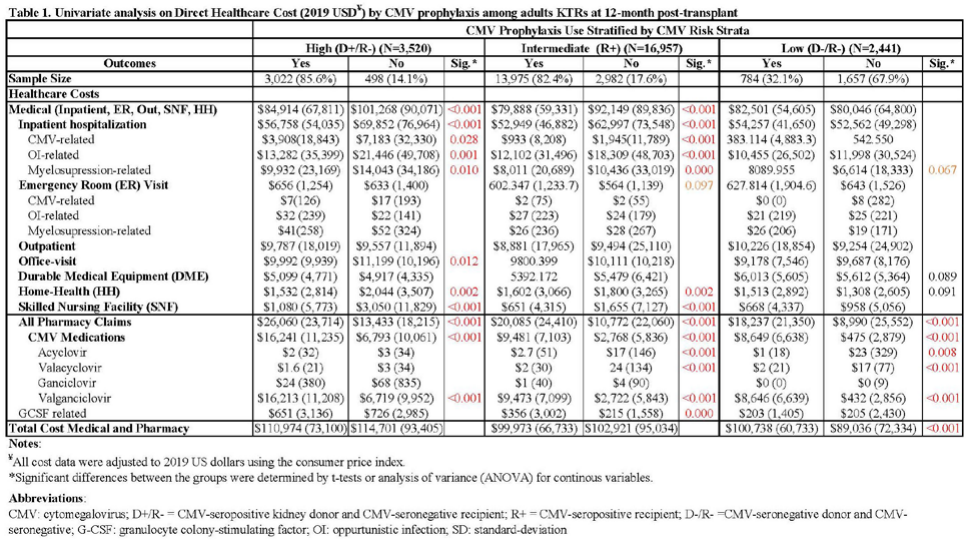

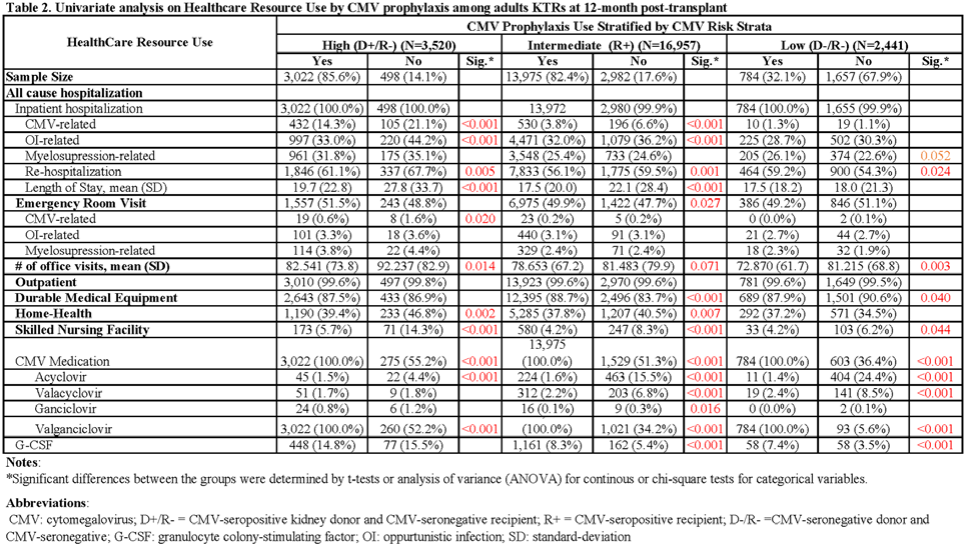

**Disclosures:**

**Amit D. Raval, PhD**, **Merck and Co., Inc.** (Employee) **Yuexin Tang, PhD**, **JnJ** (Other Financial or Material Support, Spouse’s employment)**Merck & Co., Inc.** (Employee, Shareholder)

